# Parents’ knowledge, awareness and attitudes of cord blood donation and banking options: an integrative review

**DOI:** 10.1186/s12884-018-2024-6

**Published:** 2018-10-10

**Authors:** Lisa Peberdy, Jeanine Young, Debbie Louise Massey, Lauren Kearney

**Affiliations:** 1 School of Nursing, Midwifery and Paramedicine, University of the Sunshine Coast, Locked Bag 4, Maroochydore DC, QLD 4558 Australia; 2 Sunshine Coast Hospital and Health Service, Maroochydore DC, Queensland Australia

**Keywords:** Cord blood banking, Cord blood donation, Cord blood stem cells, Women’s knowledge, Expectant parents’ knowledge, Information sources

## Abstract

**Background:**

For over 25 years cord blood has been used as an alternative to bone marrow for therapeutic use in conditions of the blood, immune system and metabolic disorders. Parents can decide if they would like to privately store their infant’s cord blood for later use if needed or to publicly donate it. Parents need to be aware of the options that exist for their infant’s cord blood and have access to the relevant information to inform their choice. The aim of this paper is to identify parent’s knowledge and awareness of cord blood donation, private banking options and stem cell use, and parent sources and preferred sources of this information.

**Methods:**

An integrative review was conducted using several electronic databases to identify papers on parents’ knowledge, attitudes and attitudes towards umbilical cord blood donation and banking. The CASP tool was used to determine validity and quality of the studies included in the review.

**Results:**

The search of the international literature identified 25 papers which met review inclusion criteria. This integrative review identified parents’ knowledge of cord banking and/or donation as low, with awareness of cord blood banking options greater than knowledge. Parents were found to have positive attitudes towards cord blood donation including awareness of the value of cord blood and its uses, with the option considered to be an ethical and altruistic choice. Knowledge on cord blood use were mixed; many studies’ participants did not correctly identify uses. Information sources for parents on cord blood was found to be varied, fragmented and inconsistent. Health professionals were identified as the preferred source of information on cord blood banking for parents.

**Conclusions:**

This integrative review has identified that further research should focus on identifying information that expectant parents require to assist them to make informed choices around cord blood banking; and identifying barriers present for health professionals providing evidence based information on cord blood use and banking options.

**Electronic supplementary material:**

The online version of this article (10.1186/s12884-018-2024-6) contains supplementary material, which is available to authorized users.

## Background

For over 25 years cord blood has been used as an alternative to bone marrow for therapeutic use in conditions of the blood, immune system and metabolic disorders [1]. Cord blood is now one of the main haematopoietic stem cell sources [2]. Umbilical cord blood banking is the process of collecting and storing umbilical cord blood, in the immediate period after the birth of a baby. Cord blood can be collected and stored either publicly or privately.

Public cord blood banks operate in all developed countries, and within most developing countries. By 2014, the international cord blood banking network comprised over 160 public cord blood banks in 36 countries, with over 731,000 umbilical cord blood units stored [3]. Public cord blood banks collect, transport, process, test and store cord blood units which have been altruistically donated for allogeneic use, at no financial cost to the donating parents [4–9]. The donated cord blood unit is not reserved for the use of the donating family, who relinquish their rights of ownership of the blood to the banking facility [10].

Private cord blood banks charge parents a fee for the collection, processing and storage of their infant’s cord blood for exclusive autologous or family use [4, 8, 9, 11, 12]. Some private cord blood banks now also store cord tissue.

Parents can decide if they would like to privately store their infant’s cord blood for later use if needed, publicly donate it, defer cord clamping to allow their infant to receive optimal volumes of cord blood at birth or to discard the remaining cord blood with the placenta after birth. Parents need to be aware of the options that exist for their infant’s cord blood and have access to the relevant information to inform their choice. Parents’ knowledge and understanding of cord blood banking and donation has been reported to be low and little is known about their source of information on this topic and the quality of the information provided [13–15]. Thus, accuracy of information is difficult to assess and there is limited understanding of how parents use this information to inform their decision making about cord blood banking and donation.

## Methods

### Aim

In this integrative review, we aimed to identify a) parent’s knowledge and awareness relating to cord blood donation, private banking options and stem cell use; b) sources of information received, and c) parents’ perceptions of appropriate sources and personnel to provide this information. The rationale for the integrative review was to identify gaps in knowledge and to provide direction for the development of antenatal education frameworks for parents in this important and evolving field of cord blood banking and cord blood use.

### Methodology

The integrative method chosen for this review allowed for rigorous evaluation of the strength of the evidence from a combination of diverse methodologies (Whittmore and Knafi 2005), and identification of gaps in the literature and areas for further research [16]. The five stages model [17] of problem identification, literature search, data evaluation, data analysis, and presentation [16], was used as a framework to guide this integrative review.

### Literature search

Databases searched included PubMed, Scopus, MIDIRS, CINAHL and Google Scholar using search terms: cord blood banking, cord blood donation, cord blood stem cells, women’s knowledge, expectant parents’ knowledge, parent/parental knowledge, sources. Publication date limits were set between 1991 and July 2017. Cord blood banking was reported to have commenced in 1991 [18]; no papers were found on this topic prior to 1998.

### Inclusion and exclusion criteria

Inclusion criteria for the review consisted of original research studies that investigated and reported parents’ knowledge, awareness and attitudes of cord blood donation and banking options, written in the English language. The initial search was conducted by the first author who identified the potential studies for inclusion based on title and abstract, with all papers for inclusion discussed and agreed upon by co-authors.

Exclusion criteria included papers not available in the English language, discussion papers, papers reporting on knowledge and awareness of embryonic stem cells, and papers which reported only on women’s choices and reasons for choice.

**Fig. 1:**
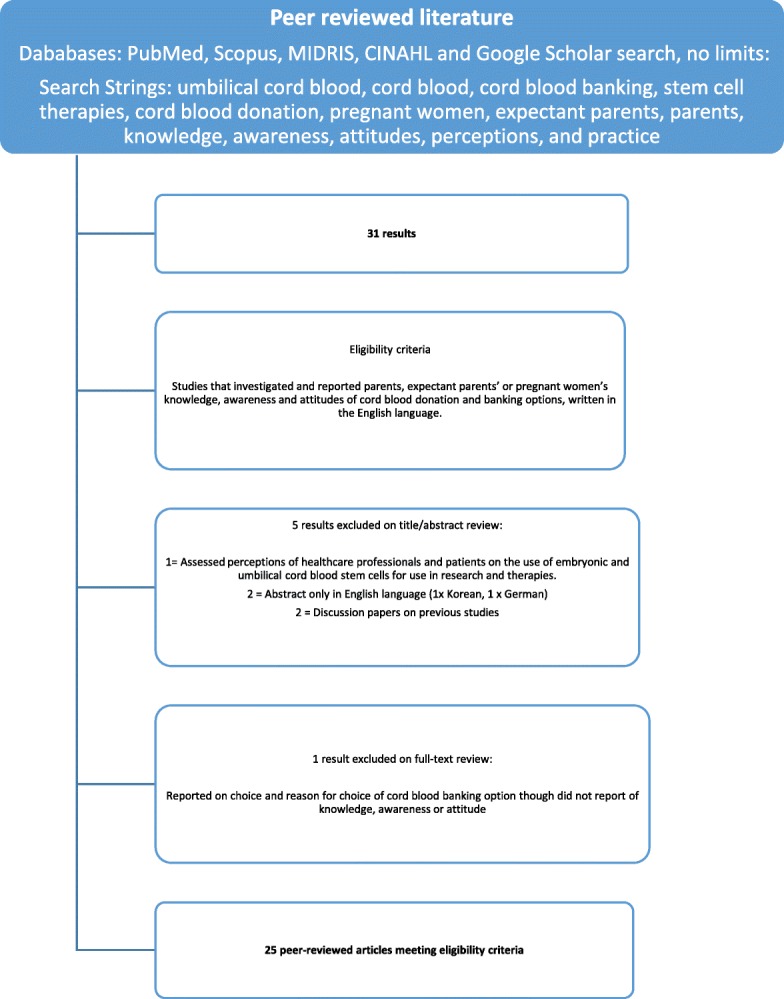
Peer Reviewed Literature Screening and Inclusion Process

Figure 1 details the structured search conducted, including the search strategy and inclusion process applied to the peer reviewed literature which was included in this integrative review.

### Data evaluation

Each article was read and summarised to identify the key points and common themes. Following the identification of these, the similarities and differences between studies were compared. Critical Appraisal Skills Programme (CASP) tools appropriate for the study designs were used to determine the quality of the studies [19]. Quantitative studies were assessed using the CASP Cohort Study Checklist (see Additional file 1). Qualitative and mixed methods studies were assessed using the CASP Qualitative Checklist (see Additional file 2). No papers were excluded because of their validity or quality.

### Data analysis

A total of 31 articles were retrieved that provided description relating to parents’, expectant parents’ or pregnant women’s knowledge and awareness of cord blood banking and donation. Only one paper retrieved also explored pregnant women’s and/or expectant parents’ knowledge and awareness of cord tissue banking [20]. Six papers were excluded because they did not meet the inclusion criteria, or aims of this integrative review [11, 21–25].

Thematic analysis [26] was used to identify emerging domains and themes in the literature, with three common domains identified: cord blood banking options, cord blood uses, and information sources.

## Findings

This search of the international literature identified 25 papers of parents, pregnant women’s and expectant parents’ knowledge and awareness of cord blood banking and donation which met the review inclusion criteria [13–15, 18, 20, 27–46]. Studies selected for inclusion in the review included empirical studies using qualitative (*n* = 5), quantitative (*n* = 18) and mixed methodologies (*n* = 2).

Overall, papers exploring pregnant women’s and expectant parents’ knowledge and awareness of cord blood donation and banking, were conducted in 15 countries: North America and Canada [13, 15, 18, 27, 28, 31], Europe and the United Kingdom [14, 29, 30, 32, 34, 36, 41, 42, 46], Australia [40], Asia and the Middle East [33, 35, 37, 43–45], Africa [38, 39] and one international study involving countries in Europe, Asia, Australasia, the Americas and Africa [20].

This integrative review included descriptive quantitative studies predominantly using survey designs [13–15, 20, 22, 30, 33–36, 39–43, 46]; qualitative studies predominantly comprising focus groups and interviews [18, 27–29, 31, 37]; or mixed method approaches using a survey design with interviews and focus groups [32, 38] to describe knowledge, awareness and attitudes of cord blood donation and banking options. Table 1 summarises the papers included in this review.

**Table 1:** Overview of papers included in the review (Knowledge, awareness, attitude, information source, public donation, private banking)

No.	Author/Year	Aim	Country Setting	Sample Inclusion	Design	Findings	Limitations
1	Mathevic & Erjavec (2016)	To investigate awareness, level of knowledge, attitudes and information sources of pregnant women and hospital maternity staff about cord blood banking.	Croatia 2 Maternity OPD	960 women 96% response rate	Quantitative Questionnaire	*Overall:* Preference of voluntary donation. One-third opted for private donation. 50% pregnant women who were not planning on CCB this pregnancy most often stated insufficient knowledge and too much paperwork *Knowledge and awareness:* Increases with age, education level and pregnancy duration. Majority unaware of practical information. *Information sources:* Media main source; 6% from Obs; nil from other HPs	Strength: Large sample size in two hospitals partly representative of city population. Weakness: Participant demographics representative of urban not general population, although UCB mainly performed in urban populations Validation of tool not disclosed
2	Matsumoto et al. (2016)	To investigate public opinion and knowledge about cord blood banking in Jordon.	Jordon 6 Maternity Hospitals (4 Private, 2 Public) Maternity OPD	899 women 100% return rate Convenience sample	Quantitative Questionnaire: multi choice, Likert-scale, and coded short answer format. Tool developed and administered by authors.	Overall: Positive public opinion about CBB. Most wanted more information on CBB, especially from Obs. *Knowledge & awareness* *69% reported low knowledge of CBB & transplant *77% reported low knowledge of CBD *Higher education & household income = more likely to hear/discuss CBB with Obs. *Only 7% heard about CBB from Obs *Attitudes and opinions* *CBD supported more than CBB; Higher likelihood of CBB if presented with future potential or recommended by Obs *Women with prior knowledge about CB transplants found it ethical /more willing to do CDB *Preference and information* *66% want more CBB information *71% Obs preferred information source	Refusal rate not recorded Not all questions answered fully
3	Kim et al. (2015)	To assess the knowledge & attitude of early post-partum women in Korea with regard to storage, donation & disposal of CB, & to identify factors influencing CB donation.	Korea 3 metropolitan maternity hospitals	320 early post-partum women who had stored (*n* = 109), donated (*n* = 34), discarded (*n* = 177) their cord blood. Convenience Sample	Quantitative 2 Questionnaires, yes/no answer format for knowledge assessment; 4 point scale for attitude format. Tools adapted from 2 previous studies Kim et al. (2009) and Lee (2006)	Overall: CBD decided earlier than CBB. Mass media most influential factor for CBD Reasons for CBB/CBD *93% CBB - as insurance for baby. *73% CBD - due to unlimited uses of CB *Knowledge and Attitude of CB use* *Higher knowledge and positive attitude towards CBB & CBD increased likelihood of CBD *CB Education* *Women who used CBD were encouraged by media *44.2% who CBB and 12% who CBD were educated HP	Lacked thorough examination on delivery of CB education Some participants believed they were educated on CBB by HP but they were CBB employees working in the hospitals
4	Bioinformant (2014)	To determine the factors involved in expectant parents’ decision to privately store, publicly donate or discard their infant’s cord blood.	International: Australia, NZ, Asia, Europe, USA, Canada, Middle East, Sth America, Mexico, Central America, Caribbean, Africa	603 Expectant parents and recent parents (within 3 years) Sample method unsure	Quantitative Survey Questionnaire Branched survey. Specific questions asked of different respondent populations.	Overall: Most study participants had not been informed of CBB options by their antenatal health care professional. *Source of CB banking information:* Obs (35%), Family & friends (35%), ANC (14%). 45%: Information from CBB was influential in their decision. 30%: Obs significantly influential in parent decision. 77%: did not CBB as unaware of option. 62%: Obs did not mention CBB. 63%: ANC did not mention cord blood banking.	Analytical strategy was not described
5	Jordens et al. (2014)	To explore awareness and understanding of cord blood banking among Australian women, and the effect of education of planned choices on the disposal of cord blood.	Australia, NSW 14 public and private antenatal clinics and classes in maternity hospitals in metropolitan (*n* = 8), regional (*n* = 4) and rural (*n* = 2) [included 3 hospitals that facilitate CB donation]	1873 Pregnant women (> 24 wks gestation, low risk) Target *n* = 2050 Response rate = 87% Purposive Sampling	Quantitative Self -administered Questionnaire: multi-choiceformat. (modified version of Fernandez et al., 2003)	Overall: Most women wanted information from ANC provider Many respondents were aware of CBB. CBB education increased intention to CBB / CBD *Awareness* *71% indicated awareness of CBB; more likely to know of CBB vs CBD Source of CB banking information*Hospital print information (43%); print media (22%); ANC (21%), TV / radio (19%), family/relatives (17%) *Decisions about CBB* *After receiving CBB basic information, proportion who indicated they would CBB or CBD increased from 30 to 68%. *CBB preferences and beliefs* *Only 13% had been asked about CBB or CBD prior to commencing survey. *93%: CBB and CBD information during pregnancy should be given by ANC giver.	Only 1 State of Australia sampled; not representative of national population Awareness, not knowledge was reported
6	Alexander et al. (2014)	To determine awareness of CB donation and banking among pregnant women.	Nigeria 1 tertiary university teaching hospital, ANC	302 Pregnant women Convenience sampling	Quantitative Structured Questionnaires	Overall: Awareness of CBD and CBB among pregnant women is low, with media the main source of information. *Awareness* *Only 19% aware of CB due to the absence of CBB and CBD in Nigeria. *Information sources* *Hospitals (30%); Media (39%), Friends (24%), Internet (7%)	CBB and CBD not available in the Country so may contribute to low awareness.
7	Karagiorgou et al. (2014)	To analyse the attitudes and knowledge of Greek citizens with high reproductive capacity (aged 18–24 years) about cord blood banking and therapies.	Greece 5 Greek cities, 2 Greek island communities.	1019 Public citizens; 292 parents Response rate = 100% of approached target population Random Sampling	Quantitative Standard anonymous multi-choice questionnaires	Findings from parents only reported here Overall: High CBB awareness level, with almost half informed by a HP *Knowledge and attitudes about CBB* *80% knew of CBB; 83% aware of CB uses; 87% positive about CBB *Information quality* *48% stated main source of CBB information, 43% of CB use information came from HP. Future attitudes *53% preferred CBD vs 47% preferred CBB for future use.	Focused on general population of childbearing age. Did not clearly represent pregnant women or expectant couples. Awareness not knowledge reported.
8	Vijayalakshmi, (2013)	To assess antenatal mothers’ knowledge regarding cord blood collection and storage. To find an association between knowledge and demographics on cord blood collection and storage.	India 1 regional hospital’s ANC	100 Antenatal mothers Non-probability Purposive sampling	Quantitative Questionnaires	Overall: 95% had poor knowledge of CBB and collection. *Significant association between knowledge scores and demographics (live birth, abortion, death, place of residence, type of family and membership to any organization) was found *Age, religion, gravida, para, education, occupation, income, newspaper and magazine subscription showed no correlation with knowledge score	Minimal information on knowledge questions asked Minimal analysis of findings presented Survey tool not validated
9	Meissner-Roloff & Pepper (2013)	To assess the extent of public support for the establishment of a public cord blood bank.	South Africa 1 urban university hospital, ANC	217 Mothers Convenience sampling	Mixed methods Qualitative Interview and education Quantitative Anonymous Questionnaires Survey tools validated	Overall: Study revealed positive support for a public CB bank in South Africa *Willingness to donate placenta and CB* *80% supported placental donation, while 2.5% unwilling to donate placenta would do CBD *78% supported a public bank; 78% willing to have HIV testing for CBD process *Knowledge of CB stem cells* *70% unaware of stem cells prior to education session*;* 94% opinion that stem cells could treat blood disorders *Influence of Age* *Younger women more willing to donate placentas than older women (84% v 77%), more likely support CBD (92% v 82%)	Centre specialized in high risk pregnancies; participants may have had better access to, and received more, information than rest of population attending other clinics
10	Padmavathi (2013)	To assess stem cell and CB banking knowledge among antenatal mothers before and after a structured teaching program. To assess the effectiveness of structured teaching program on cord blood banking and stem cell knowledge among antenatal mothers.	India 1 district maternity hospital, ANC	30 Antenatal mothers Purposive sampling	Qualitative Structured interviews pre and post education Post education interviews attended 7 days following education	Overall: Results suggest a structured teaching program was effective and increased ANC mothers’ knowledge on stem cells and CB. *Pre-test Knowledge* *57% had poor knowledge; 43% had average knowledge. *Post-test knowledge* *70% had good knowledge; 30% had average knowledge. *Mean post-test knowledge higher (21.9%) then pre-test knowledge (10.2%).	Unclear of education content in teaching session and how knowledge was assessed Unclear if same interview questions used pre and post education.
11	Screnci et al. (2012)	To explore knowledge about CB stem cells, and preferences for donation or private banking and the motivation behind the decision.	Italy University of Rome, ANC	239 pregnant women before CB education given Surveys distributed *n* = 300 Response rate = 80% [298 female blood donors] Convenience Sampling 100 mothers who had donated CB (for verification of donation motivation)	Quantitative Anonymous Questionnaires	Findings reported for pregnant women only. Overall: Large support for CBD suggests CBB is not an obstacle to expansion of CBD. HP and institutions should provide CBB information. *Knowledge of CB* *93% general knowledge; 42% probability of clinical use; 31% therapeutic uses; 58% difference CBD Vs CBB; 71% donation criteria *CBD awareness* *95% aware of CBD *Information source* *42% Obs; 25% internet *CB choice* (*n* = 215) *61% would CBD, 56% had altruistic and other reasons; *7% would CBB, 73% would do so to safeguard future *32% would discard CB, logistics (28%), lack of interest (28%)	Sample from one Institution only so may not be generalised Survey tool not validated
12	Shin et al. (2011)	To investigate the knowledge of CB and attitudes towards CB banking among well educated, high-potential donors.	Korea 1 Maternity hospital	863 pregnant women attending antenatal classes which did not consist of CB banking education component Convenience sampling Surveys distributed = 1430 Response = 60.3%	Quantitative Questionnaires Questionnaire adapted and enhanced from 3 previous studies (Fernandez et al. 2003,Perlow et al. 2006, Fox et al. 2007)	Overall: Minimal level of knowledge was recorded. Obs have insignificant role in disseminating knowledge *Knowledge* 57% correctly answered CB current use and limitations *CB collection reason* *CBD: Altruism most common reason (94%) *Safeguard for future was most common reason for CBB (75%) *Most common reason for no CB collection was inconvenience of consent and medical questionnaire *CB Donation motivation* *54% of CBD were blood donors *Source of information* *88% received CBD information; most common sources CBD of information was media/internet (37%) and brochures (31%). * 2% and 4% learnt about CBD and CBB respectively from Obs. *97% received CBB information; most common CBB information source was advertisements (38%) and media/internet (36%).	Only highly educated,urban women who received antenatal care and education were included. Results may not be generalized. Survey Tool not validated.
13	Manegold et al. (2011)	To explore the attitudes of donating parents towards public and private CB banking.	Switzerland Public CB bank	300 Recent Swiss, western and eastern European public CB donors. Purposive Sampling Surveys distributed = 621 Response rate = 48.3%	Quantitative Standardised anonymous questionnaire 20 multi-choice and open ended questions	Overall: Motivation for private or hybrid CB banking is low in this population. *Source of CBD information* *54% from HP *22% from more than 1 source: family, friends and media *34% actively sought CBD information *CBD* vs CBB Options *2% would CBB for next infant *27% did not know of CBB *69% opted for CBD due to altruism and cost of CBB	84% of the open questions were unanswered Only donors whose CB was accepted for storage were included in study May not be generalized to the entire donor population Survey tool not validated
14	Katz et al. (2011) Europe	To explore pregnant women’s awareness of CB stem cells and their attitude towards banking.	5 European countries: France, Germany, Italy, Spain, United Kingdom. 6 urban maternity hospital antenatal clinics with over 1000 births per annum (Germany = 2 antenatal classes in lieu of clinic)	1620 Pregnant women who had not previously enrolled in a CB banking program. France *n* = 318 UK *n* = 290 Germany *n* = 313 Spain *n* = 323 Italy *n* = 376 Purposive Sampling	Quantitative Anonymous self-directed multi-choice questionnaire	Overall: Study revealed strong preference for CBD. Attitudes were not an obstacle to CBB. *CB Information and knowledge* *79.4% declared poor CBB knowledge. *59.6% received information via mass media and internet. *20% received information from HP. *91.6% believed they should be systematically informed. *CB banking choices* *89% would collect CB; 11% would discard CB; 77% would CBD; 12% would CBB; 12% would store in hybrid bank *Choice for CBD* *59% said altruism; 30% believe a duty to donate *24% would change birth hospital in order to be able to CBD *Choice for CBB* *12% would CBB; 51% of these women would do so due to possible future medical research therapies *16% would do so for insurance reasons	Ethnic breakdown was not reported. Data collection differed across sites: German questionnaires conducted in antenatal classes not clinics as in other 4 countries CBB not available in 3 countries at time of study (France, Italy and Spain) Knowledge not awareness reported. Survey tool validated
15	Suen et al. (2011)	To assess knowledge of private cord blood banking among pregnant women	Hong Kong 2 large public maternity units	1866 Pregnant women accessing antenatal clinic. Surveys distributed = 2000 Response rate = 93.3% Convenience Sampling	Quantitative Cross-sectional self-administered questionnaire Survey validated	Overall: Study revealed inadequate knowledge on CBB and use. *Understanding* *78.2% reported no understanding of likelihood CBB use *Awareness* *Only 58.5% were aware of CB use for childhood leukemia *Knowledge* *20.3% knew of CB availability from public CB banks *Preferred source of CBB information* *44% stated Dr.; 32% stated CBB staff *22% stated unsure who to receive information from; 7% stated N/MWs Government involvement * 89% wanted more promotion and education on CBB	Sampling limited to public patients who did not have the option of CBB unless indicated for medical reasons.
16	Salvaterra et al. (2010)	To analyze knowledge, comprehension, opinions, attitudes and choices related to cord blood donation of pregnant women, future parents, donors, midwives, obstetricians/ gynaecologists. To compare preferences of public versus private banking.	Italy Hospital, community & academic sector participation	Pregnant women, future parents and donors (*n* = 30) 32 antenatal health care providers consisting of: 10 community midwives 12 hospital midwives 10 obstetricians (public and private) Multiple sampling methods	Mixed methods using participatory approach with establishment of a taskforce and public multidisciplinary round table Focus groups; (max. n = 10 participants, led by 2 psychologists) Self -administered questionnaires at completion of focus groups (*n* = 20)	Findings reported from pregnant women, future parents and donor perspectives: Overall: *CBD considered a gift of moral and social value; Participants would CBD for altruistic purposes. *CBB was associated with egoism and fraud. *100% wanted more information and clear procedures on CBB. *100% stated HP should be educated on CBB/CBD and inform future parents during pregnancy *70% (14/20) reported poor knowledge of CBD	Included only those in an urban setting and didn’t include any minority groups. Few knowledge questions; most opinion based. Small sample sizes allowed for limited between group comparisons Researchers developed own assessment tool Knowledge not reported
17	Rucinski et al. (2010)	To explore the knowledge, attitudes, beliefs and practices regarding cord blood donation among Hispanic and non-Hispanic black women.	United States of America 1 Community Health Centre and 1 Community Hospital in Chicago, Illinois	41 Hispanic and non-Hispanic pregnant black women, or who had given birth in the last 12 months, > 18 yrs., had received antenatal care by the 2nd trimester; did not have any religious objections to donation. Purposive sampling.	Qualitative 5 Focus groups: 1 Hispanic (English) *n* = 5 1 Hispanic (Spanish) *n* = 9 3 non-Hispanic *n* = 8/9/10	Overall: Most not aware of, what it involved, or the value of, CBD for treatment and research. Participants believed that Drs provide CBD information Initial analysis did not reveal strong ethnic difference in knowledge or attitudes towards CBD. *Knowledge/Awareness* *Participants who reported awareness of saving CB, was in reference to CBB not CBD. *Participants reported confusion between CBD and CBB options. *Information needs and sources* *Those who had birthed expressed concern that they had’t been informed by HP on CBD option *Many wanted CBD info from their Dr. due to trust/respect in Dr. being source of factual information and perceived ability to answer questions on topic. *Some parents reported Dr. indifference on topic and Dr. failure to spend time providing health related answers to questions which reduced faith that Drs were reliable source of information.	Very specific inclusion criteria so results could not be generalized to the wider population.
18	Palten & Dudenhausen (2010)	To evaluate the correlation between German-speaking women’s knowledge of cord blood banking and their level of education.	Germany (Perlow, 2006) 1 obstetric hospital in Berlin, 3 ANC	300 Pregnant women over the age of 18 years in their 3rd trimester Surveys distributed = 313 Response rate = 96% Quota Sampling: to gain comparative number to Fox et al. (2007) study	Quantitative Multi-choice response Questionnaire	Overall: Women were poorly educated about CB storage usefulness, costs and methods. *Education* *35% well educated (University degree). *Women with higher education level had read more CBB information *Knowledge* * 50–65% were unaware of CB treatable illnesses *Source of CB information* *74%: reading material and commercials. *59%: material by private CBB. *26%: public CBD banks. *CB discussion with obstetrician* *5% discussed it with Obs; 1% had it raised by Obs	Language interpreted tool used by Fox et al. (2007), although cultural and health system differences make comparisons of findings difficult. Awareness not knowledge reported.
19	Dinc & Sahin (2009)	To determine pregnant women’s knowledge and attitudes towards stem cells and cord blood banking in Instanbul.	Turkey 2 Antenatal clinics: 1 in a University Medical Centre, 1 in a Family Planning Centre.	334 Pregnant women accessing antenatal clinic in Instanbul. Convenience Sampling	Qualitative Exploratory descriptive study of Interviews: yes/no and open ended questions	Overall: Women with a higher education had higher levels of knowledge about CB and stem cells. Most had a lack of knowledge on the topics and wanted more information from HP. *Knowledge* *Only 26.9% aware of CB and stem cells. *Source of CB information* *72% stated media; 28% stated Obs *Preferred source of information* *79% stated Obs; 21% stated N/MW *Main reasons for CBB* *48.9% stated possible future need *22% it is beneficial; 10% future regret *8% insurance for child *Main reasons against CBB* *68.7% not necessary; 21% limited information	Select sample of women in 2 antenatal clinics in1 location so may not be generalized to the rest of the population. Awareness not knowledge reported.
20	Fox et al. (2007)	To evaluate patient understanding of cord blood banking.	United States of America 1 large Obstetric Hospital, New York with access to public and private CB banking, ANC	325 pregnant women Quota sampling Surveys distributed =724 Response rate = 44.9%	Quantitative Anonymous multi-choice questionnaire	Overall: Women had very poor understanding of CB uses and banking. *Education status* 94% completed undergraduate degree 58% completed post graduate degree. *Awareness* *54.4% unaware of medical conditions treatable with CB. *Main CB Information source* *86.5%: private CBB literature *29.2%: Public CBD banks literature *36.9%: Discussion with Obs though not stated who initiated the conversation. *Reasons for private CBB* *83%: protect infant in future	Survey conducted in early pregnancy. Only 45% of surveys completed so may indicate a bias of results. Study did not examine the extent of the women’s knowledge of CBB.
21	Perlow (2006)	To determine patients’ knowledge of cord blood banking.	United States of America 1 Obstetric Medical Centre Phoenix, Arizona.	425 Pregnant women attending for antenatal consultation, or ultrasound. Convenience Sampling	Quantitative Convenience Sampling 2. part questionnaire: 1. Awareness 157 (37%) unaware of CB banking. Completed part 1 only. 2. Knowledge 268 (63%) completed part 1&2.	Overall: Patients poorly informed about CBB (74%, 315/425). Few receive CB education from HP. Lack of knowledge and expense CBB barriers. *Awareness of CBB* *63% were aware. Remainder excluded from part 2 of study. *Women with lower education less likely to be aware than women with a University degree (22% v 78%). * Women under age 25 less likely to be aware (53% v 68%). *Ethnic women had less awareness then Caucasian women. *Knowledge of CBB* *74% stated minimally informed. *3% stated extremely knowledgeable on the subject. *Source of CBB information* *53% informed by media; 17.5% informed by Dr.; 8.2% informed by other HP. *Barriers to CBB* *Cost (30%); low knowledge (31%), misinformation on who could use CB (50%).	Addressed private CBB only. Conducted in one location only so may not be representative of the general population. Lack of cultural diversity, small numbers of Native and African Americans in the survey. Last two questions of the survey were not completed by all participants.
22	Danzer et al. (2003)	To evaluate the attitudes of mothers towards cord blood donation for therapeutic use 6 months post donation.	Switzerland 1 University Hospital with a CB collection centre	78 Women 6 months post- partum who donated cord blood Purposive Sampling Response rate = 59.5% (Total surveys distributed = 131)	Quantitative Survey Standardized anonymous questionnaires Multi-choice format, with 1 open ended question.	Overall: A High degree of satisfaction with CBD. *Responses regarding CBD* 100% believed CBD was ethical *96.1% would CBD again *74.8% emotionally satisfied about CBD *Original source of CBD information* *81.3% from their Dr.; 18.7% from media and friends. *No significant association between information source and decision to donate again. Open ended question comments *8 women supported importance of CB collection centres; 5 expressed concern for improper use, 2 expressed concern donated CB may not be available for own child.	Women from one Institution only were surveyed. A total of 131 were sent surveys however, 40.5% did not respond.
23	Fernandez et al. (2003)	To examine pregnant women’s knowledge and attitudes regarding CB banking, which maybe used in the development of policies and procedures for public and private CB banking?	Canada 1 Regional Women’s Hospital	443 English speaking pregnant women attending antenatal clinic. Response rate = 68% (Total surveys distributed = 650) Convenience Sampling	Quantitative Questionnaires developed by Authors	Overall: Most women were supportive of CBD for transplantation and research. *Knowledge* *72% reported poor or very poor CBB knowledge (*n* = 310) 25% overestimated risk of a child requiring a stem cell transplant *Preferred CB education source* *66% HP; 68% Dr.; 70% ANC. *CB Banking option* *14% would choose CBB due to a good investment. *86% would choose to CBD due to altruism.	High proportion of participants were university or college educated. Little ethnic diversity in group. No established public or private CB banks in the area at the time the study was conducted.
24	Sugarman et al. (2002)	To evaluate the informed consent process for cord blood donation.	United States of America 2 CB Collection centres associated with a Public CB Bank.	170 Pregnant women in the 3rd trimester who had consented to cord blood donation Convenience Sampling	Qualitative Telephone interviews	Overall: Women were satisfied with consent process (96.9%), most (98.8%) would donate again, though did not seem to know about alternatives to CBD. *Other responses to CBD process* *Only 32.9% understood they had the option not to have CB collected. *Only 55.3% understood the option of CBB. 78.8% incorrectly thought they could donate CB to a specific recipient. Incorrect endorsement of CBD *Diagnosis of genetic disease of infant (92.9%) and mother (88.2%) * Diagnosis of infectious disease of infant (88.2%) and mother (87.1%) *Protection for infant (48.8%)	Sample limited to those who had consented to CBD at 1 public bank. Understanding of CBD and uses may differ in women who chose not to CBD and where CBB is an option. Interviews were conducted 1 month post -partum so information previously conveyed and understood may have been forgotten.
25	Sugarman et al. (1998).	To learn about pregnant women’s concerns regarding CB collection and banking in order to establish a comprehensive recruitment and informed consent process for donation.	United States of America 3 antenatal clinics (1 private, 2 public) affiliated with CB collection centres.	19 Pregnant women in their 2nd & 3rd trimester Purposive sampling	Qualitative Focus group discussions	Overall: 100% indicated they lacked sufficient or substantial prior knowledge of CB technology. Desire for more information about collection, storage and use of CB, especially difference between CBB and CBD was identified. *CB education* *100% believed in importance of CBB education including collection, storage and use. *Earlier the education was provided promoted a feeling of choice. *CB education should be in various formats: clinic pamphlets/posters, parenting magazines, information hotlines, television advertisements & reports, ANC. *Safety of mother and infant* *Important to inform women that CB collection does not alter the birth process. *Reasons for CBD* *Altruism was main reason *Influence of others may give cause for more likely CBD.	Findings context specific, not able to be generalized to broader population.

Key: *CB* Cord blood, *CBB* Private cord blood banking, *CBD* Public cord Blood Donation, *Obs* Obstetrician, *N/MW* Nurse/Midwife, *Dr* Physician, *HP* Health Professional, *ANC* Antenatal Class, *OPD* Outpatient Clinic

Three domains pertaining to pregnant parents’ knowledge, awareness and attitudes were identified: a) cord blood banking and donation; b) cord blood use; and c) cord blood information sources and preferred information sources. Cord blood banking and donation options encapsulated three themes: knowledge, awareness and attitudes. The second domain, cord blood use, comprised two themes: knowledge and awareness. The final domain, information sources, was also divided into two themes: actual sources and preferred sources of information on cord blood banking and donation.

### Cord blood banking and donation

Seven papers investigated and reported on cord blood banking awareness [13, 15, 31, 39–41, 46]. Four studies reported a high level of awareness, with around 70% of participants reporting awareness of the topic [15, 40, 41, 46]. Women of lower education levels, age 25 years or less, or of an ethnic minority background were factors associated with less awareness of banking and donation [15, 40].

Three papers reported low awareness of cord blood banking and donation [13, 31, 39]. Participants who had heard about cord blood banking expressed considerable confusion between public and private banking options [31], with cord blood donation having the least awareness reported in North America [13, 31].

Thirteen studies reported on cord blood banking and donation knowledge [14, 15, 18, 27, 28, 32–34, 37, 41, 43–46], with most studies assessing knowledge by participant self-report, as opposed to knowledge being measured by assessment of associated facts. Ten studies identified parent-reported suboptimal knowledge about collection and storage options for cord blood [15, 18, 27, 28, 32–34, 37, 43, 44], and of parents being minimally informed about cord blood banking and donation options [15, 28, 32–34, 37, 44, 45].

Exceptions to these low knowledge findings were reported by four studies, with more than 70% of participants of three studies reported to be knowledgeable about cord blood banking and donation [14, 41, 46]. Findings from early postpartum women (*n* = 320) surveyed by Kim et al. (2015) on their knowledge and attitudes of storage, donation and disposal of cord blood suggested that a high level of knowledge about cord blood was associated with women opting for cord blood donation.

Ten papers investigated parents’ attitudes towards cord blood banking and donation with samples including pregnant women, expectant parents and new parents [14, 28, 29, 32, 34, 35, 41, 42, 44, 46]. Overall, the findings from these studies indicated that parents were more inclined to support donation than private cord blood banking [14, 28, 32, 34, 35, 42, 45]. Key themes of parent attitudes towards donation and storage of cord blood included altruism, ethical practice, duty to society and insurance for the baby. Only one paper reported low regard for altruism or public benefit surrounding cord blood donation, however this may be attributed to lack of awareness of cord blood donation as public cord blood banking was not available at the time of this study’s data collection [45].

Several papers found parents to be positive towards cord blood banking [29, 41, 44, 45]. Reasons given for private cord blood banking included insurance for their baby [44], the cord blood may be needed in the future and they may have future regret of not storing their baby’s cord blood [29].

### Cord blood use

Five papers reported on cord blood use awareness [13, 31, 38, 41, 46], with only one paper reporting high awareness, which included participants who were already parents [41]. Three studies used mixed methods and reported that considerable proportions of the parent population had relatively low awareness relating to uses of cord blood [13, 31, 38].

Nine papers reported knowledge of cord blood use [13, 27–30, 33, 35, 36, 46] and knowledge deficits were identified. Treatment of blood cancers was the most commonly known use of cord blood [13, 29, 30, 35], with over 50% of participants correct in their responses in studies by Fox and colleagues (*n* = 70%) [14] and Palten and Dudenhausen (50–65%) [26]. Limited knowledge was reported for other uses [13, 30, 36], including the likelihood of use of cord blood stem cells [28, 33]. Matijevic and Erjavec (2016) reported 95% of participants in their study self-reported knowledge of cord blood treatments as either insufficient or basic [46].

### Cord blood information

#### Source of information

Source of cord blood banking information was investigated by 16 of the reviewed papers [13–15, 20, 28, 30, 31, 34–36, 39, 40, 42, 44–46]. The main sources of parent information were hospitals; health professionals, including antenatal classes; media and magazines; cord blood banks; and family and friends. Table 2 summaries the sources of information reported in the studies reviewed.

Six authors reported health professionals and/or antenatal classes were the main source of information on cord blood banking [14, 20, 36, 41, 42, 44], with a further two authors reporting these were the second most common sources [39, 40]. Health professionals, particularly doctors, were identified as important informers of cord blood banking options [20, 36, 42, 45]. Receiving this information from a health professional significantly influenced the parental decision to store cord blood [20].

Four authors reported low numbers of participants had received cord blood information from health professionals [15, 34, 35, 45, 46], and a further study found that participants had to actively enquire in order to receive information on cord blood donation [14].

Print and electronic (including internet) media and advertising were the main information source of cord blood banking reported in six studies [15, 30, 34, 35, 39, 46], and was the second most common source in two further papers [36, 40] after health professionals [36] and private cord blood banks [40].

Four studies listed cord blood banks as a source of cord blood banking information [13, 20, 30, 40], with Jordens and colleagues [36] reporting this was the main source for their participants. Private banking information was reported as a more common source of information compared to public banks [13, 30]; one study reported that almost half of their sample indicating that information from private cord blood banks was influential in their decision to store cord blood [20].

Six reports noted family and friends to be a source of information [14, 20, 36, 39, 42, 47], though only one paper stated this was their main source [20]. Three studies combined ‘family, friends and media’ as a single information source category [15, 28, 32]. These studies reported similar findings with approximately 20% of participants identifying this category as a source of cord blood banking information and an influence in their decision-making [15, 32, 38].

#### Preferred source of information

Five papers reported on participants preferred source of information on cord blood banking and donation [28, 29, 31, 33, 40, 45]. Four studies listed antenatal health professionals, including antenatal classes, as the most important and preferred source [29, 31, 33, 40, 45]. Only one paper reported cord blood banks as a preferred source of information [33]. Table 2 displays the preferred information sources reported by participants of studies included in this review.

## Discussion

Cord blood banking and donation has been an option for parents for the past quarter century, yet an understanding of knowledge and awareness of these options, and consistency of information provided to parents, remains low. This is the first integrative review to explore parents’ knowledge, awareness and attitudes towards cord blood banking and donation, and parent sources, and preferred source, of information on this topic.

This integrative review identified parents’ knowledge of cord banking and/or donation as generally low [18, 27, 28, 32–34, 37, 44–46]. Higher knowledge levels were identified where participants had previously donated cord blood and where participants had been provided with information on these options by their antenatal health care provider or in antenatal classes [14, 41, 44]. This finding highlighted the importance of providing parents with this information as part of routine antenatal education. Overall, awareness of cord blood banking options was found to be higher than knowledge in this integrative review [15, 41, 47]. Like knowledge findings, this may be attributed to the availability of information provided at birthing facilities, and the level of education of participants [15, 40, 41].

Positive attitudes towards cord blood donation among parents were found, with the option considered to be an ethical [42] and altruistic choice for parents [14, 28, 34, 35, 41]. This could be indicative that cord blood donation has a moral association, and this finding may be important when health professionals discuss this option with parents as they may feel pressure or an obligation to choose this option. Positive attitudes towards private cord blood banking were also found, with only one study reporting negative findings [32]. Participants who chose to privately store their infant’s cord blood did so because they viewed this option as an investment for future use, insurance or protection for their child or family [28, 29, 34, 35, 44]. The desire of parents to do the best for their children and provide for their future may influence their interpretation of the importance of the scientific benefit on storing cord blood stems cells for future health protection, and illustrates the emotional element frequently attached to this option.

Knowledge on cord blood use among study participants was mixed. Over 50% of participants in many of the studies could not correctly identify uses of cord blood [13, 18, 27, 29, 30, 33, 36, 46]. This lack of knowledge emphasises the uncertainty about the source and the quality of the information being provided. When knowledge was self-reported by participants, general uses for cord blood was higher than specific uses [29, 30, 36], with treatment of blood cancers the highest correct response reported [14, 26].

Awareness among parents of the value of cord blood and cord blood uses was found to be less than knowledge levels of cord blood value and use. We identified that the provision of information by health professionals greatly influenced awareness of the value of cord blood and its’ potential uses. This finding again emphasises the need for information to be provided as part of routine antenatal care.

In this integrative review, we found that there was inconsistency in information provided to parents about cord blood banking and cord blood use. This inconsistency created awareness and knowledge deficits and arguably prevents parents from making informed choices. This is an important finding; in Australia, the Health and Safety commission have identified involving consumers in health care choices is associated with better client experience and promotes client centered care [48].

Information sources for parents on cord blood was found to be varied, fragmented and inconsistent [14, 20, 35, 40]. This inconsistency of information is concerning because for parents to make informed choices about cord blood banking or donation they need appropriate, relevant, objective information that is accurate, valid, regulated and based on the latest evidence in a variety of consumer-friendly formats through trustworthy sources [49].

Health professions were identified as the preferred source of information on cord blood banking for parents [28, 29, 31, 33, 40, 45]. The views of clients are among many factors that influence change to health services [50] and it is imperative that information on cord blood banking and donation is considered as part of routine antenatal education for parents.

### Strengths and limitations of this study

The integrative approach chosen for this review of parent knowledge and awareness of cord blood banking, donation and cord blood banking, including sources and preferred sources of information, allowed for the inclusion of a diverse range of qualitative, quantitative and mixed methods studies with participant samples from nations representing most world continents. Despite the literature review being extensive, inclusive of published studies meeting eligibility criteria since cord blood banking became available in 1991, this integrative review was limited to studies published in the English language only. Different terminology and sampling descriptions (pregnant women and / or parent / couples’ knowledge) used across studies, and a lack of clarity and consistency within studies relating to study aims and methods reported, limited interpretation of some study results.

The papers included in this review varied significantly in sample size (*n* = 30 to 1873), but this may have been driven by the research approach chosen [18, 31, 32, 37]. Survey tools to measure knowledge, awareness and attitudes were poorly described or not validated in some studies [14, 32, 35–37, 43, 46], with only two studies using the same (or modified version) tool [13, 30].

Several papers reported on awareness, not knowledge, as indicated in their title or abstract [29, 30, 32, 40, 41] or on knowledge, when awareness was indicated [34]. The findings of some studies were context specific and may not be generalised [14, 18, 31, 35–37], or participants did not have access to both cord blood banking and donation which may have influenced study findings [15, 27, 28, 33, 34, 39].

### Implications for practice, education and research

In this integrative review, inconsistencies, and uncertainty in knowledge and awareness that parents have regarding cord blood use and banking options have been highlighted. These findings are indicative of the need for expectant parents to be informed of the cord blood banking options available to them by their antenatal care providers and/or at their birthing facility so that they can make an informed decision about what option is appropriate for their family circumstances. Maternity care policy and practice evolve with the emergence of new research evidence [49]; health services therefore need to be responsive to client and consumer input and needs [48] and involve clients in health care and informed decision making.

#### Research

Parent knowledge of cord blood banking options and cord blood use has been identified as poor. This integrative review identified that parents have a lack of knowledge about the options of cord blood banking and donation, and the uses of cord blood. There is lack of clarity and consistency in the information provided for parents on cord blood banking, donation and cord blood use. Future research is needed to explore health professionals’ knowledge of, and attitudes towards, cord blood banking, donation and cord blood use and how this impacts on the information that they provide to expectant parents in their care. The option of cord blood banking and donation has been available to parents for over 25 years so it is timely to investigate where the gaps in health professionals’ knowledge lie.

#### Practice

Information on cord blood banking and cord blood use is not a standard element of antenatal education and this is concerning because parents require this information to make a fully informed choice of their options regarding their infant’s cord blood following birth. We argue that there is a need for health professionals to provide accurate and evidence-based information to parents. This integrative review has demonstrated that information provision to expectant parents by health professionals on the topic of cord blood banking and donation is not a consistent part of antenatal education. Research is needed to identify and understand barriers to the information provision to parents on cord blood banking and donation, and why this important topic is not yet a standardised part of antenatal education.

#### Education

Health professionals are the parent preferred source of cord blood banking information. It is vital that health professionals are educated and informed of all aspects and elements of cord blood banking to enable them to provide appropriate information to parents. We argue that cord blood banking should be incorporated into health professional curricula and antenatal education.

## Conclusion

Cord blood banking is complex and often poorly understood by parents and health professionals. This integrative review makes an important contribution to the body of knowledge in this field by identifying knowledge, highlighting gaps and suggesting direction for future research, practice and education in relation to cord blood banking and donation and cord blood use.

Significant gaps in parents’ knowledge and awareness of cord blood banking have been identified in this review of current evidence. This is an important topic and one that requires parents to make informed and rationale choices. For this to occur, information provided needs to be accurate, objective valid, timely and appropriate, and supplied by parent preferred sources. As identified in this integrative review, currently this is not the case.

This integrative review has identified that further research should focus on identifying the information expectant parents would like to receive to assist them to make an informed choice around cord blood banking and to identifying the barriers to health professionals providing this evidence-based information on cord blood use and banking options.

## Additional files

Additional file 1: Appraisal of Quantitative studies by study design using CASP tools. CASP tool assessments of Quantitative studies listed chronologically. (DOCX 15 kb)

Additional file 2: Appraisal of Qualitative studies by study design using CASP tools. CASP tool assessments of Qualitative studies listed chronologically. (DOCX 14 kb)
