# Supplementary material for: Parents’ knowledge, awareness and attitudes of cord blood donation and banking options: an integrative review
Source: BMC Pregnancy Childbirth. 2018 Oct 10;18:395. doi: 10.1186/s12884-018-2024-6 (PMC6180365; doi:10.1186/s12884-018-2024-6)
Supplement: Supplementary file 1 — Appraisal of Quantitative studies by study design using CASP tools. CASP tool assessments of Quantitative studies listed chronologically. (DOCX 15 kb) [file 12884_2018_2024_MOESM1_ESM.docx]

**Additional file 1: Appraisal of Quantitative studies by study design using CASP tools (CASP, 2013)**

| Quantitative Studies | | | | | | | | | | | | | | |
| --- | --- | --- | --- | --- | --- | --- | --- | --- | --- | --- | --- | --- | --- | --- |
|  | Are the results of the study valid? | | | | | | | | What are the results? | | Will the results help locally? | | |  |
| Article | Did the study address a clearly focused issue? | Did the authors use appropriate methods to answer their question? | Was the cohort recruited in an acceptable way? | Was exposure measured to minimise bias? | Was outcome measured to minimise bias? | Have the authors identified all confounding factors? | Have they taken into account of the confounding factors in the design and/or analysis? | Was follow up complete? | Are results presented transparently and precisely? | Are the results plausible? | Can the results be applied to the local population? | Do the results fit with other evidence? | Does this study have direct implications for practice? | Validity score |
| Matijeviic & Erjavec (2016) | Y | Y | Y | Y | Y | Y | Y | Y | Y | Y | Y | Y | Y | 13/13 |
| Matsumoto et al (2016) | Y | Y | Y | Y | Y | Y | Y | Y | Y | Y | Y | Y | Y | 13/13 |
| Kim et al (2015) | Y | Y | Y | Y | Y | Y | Y | Y | Y | Y | Y | Y | Y | 13/13 |
| Bioinformant (2014) | Y | Y | Y | Y | N | Y | Y | Y | Y | Y | Y | Y | Y | 12/13 |
| Jordens et al (2014) | Y | Y | Y | Y | Y | Y | Y | Y | Y | Y | Y | Y | Y | 13/13 |
| Alexander et al (2014) | Y | Y | Y | Y | Y | Y | Y | Y | Y | Y | Y | Y | N | 12/13 |
| Karagiorgou et al (2014) | Y | Y | Y | Y | Y | Y | Y | Y | Y | Y | Y | Y | Y | 13/13 |
| Vijayalakshmi (2013) | Y | Y | Y | Y | Y | Y | U | Y | N | Y | Y | Y | Y | 11/13 |
| Screnci et al (2012) | Y | Y | Y | Y | Y | Y | Y | Y | Y | Y | Y | Y | Y | 13/13 |
| Shin et al (2011) | Y | Y | Y | Y | Y | Y | Y | Y | Y | Y | Y | Y | Y | 13/13 |
| Manegold et al (2011) | Y | Y | Y | Y | U | Y | Y | Y | Y | Y | Y | Y | Y | 12/13 |
| Katz et al (2011) | Y | Y | Y | Y | U | Y | Y | Y | Y | Y | Y | Y | Y | 12/13 |
| Suen et al (2011) | Y | Y | Y | N | N | N | N | Y | Y | Y | Y | Y | Y | 9/10 |
| Palten & Dudenhause (2010) | Y | Y | Y | N | N | Y | U | Y | Y | Y | Y | Y | Y | 10/10 |
| Fox et al (2007) | Y | Y | Y | Y | Y | Y | Y | Y | Y | Y | Y | Y | Y | 13/13 |
| Perlow (2006) | Y | Y | Y | Y | Y | Y | Y | Y | Y | Y | Y | Y | Y | 13/13 |
| Danzer et al (2003) | Y | Y | Y | Y | Y | Y | Y | Y | Y | Y | Y | Y | Y | 13/13 |

Y=Yes; N=No; U=Unclear

CASP. 2013. *Critical Appraisal Skills Programme (CASP): Making sense of evidence.* [Online]. <http://www.casp-uk.net/#!casp-tools-checklist/c18f8>. [Accessed April 30 2015].
