# Supplementary material for: Parents’ knowledge, awareness and attitudes of cord blood donation and banking options: an integrative review
Source: BMC Pregnancy Childbirth. 2018 Oct 10;18:395. doi: 10.1186/s12884-018-2024-6 (PMC6180365; doi:10.1186/s12884-018-2024-6)
Supplement: Supplementary file 2 — Appraisal of Qualitative studies by study design using CASP tools. CASP tool assessments of Qualitative studies listed chronologically. (DOCX 14 kb) [file 12884_2018_2024_MOESM2_ESM.docx]

**Additional file 2: Appraisal of Qualitative studies by study design using CASP tools (CASP, 2013)**

| Qualitative Studies | | | | | | | | | | | |
| --- | --- | --- | --- | --- | --- | --- | --- | --- | --- | --- | --- |
| Article | Was there a clear statement of the aims of the research? | Is qualitative methodology appropriate? | Was design appropriate to address the aims? | Was the recruitment strategy appropriate to match aims? | Were the data collected in a way that addresses the research issue? | Has the relationship between researcher and participants been adequately considered? | Have ethical issues been taken into consideration? | Was the data analysis sufficiently rigorous? | Is there a clear statement of findings? | Is the research valuable? | Validity score |
| Meissner-Roloff & Pepper (2013) | Y | Y | Y | Y | Y | N | U | Y | Y | Y | 8/10 |
| Salvaterra et al (2010) | Y | Y | Y | Y | Y | N | U | N | U | N | 5/10 |
| Padmavathi (2013) | Y | Y | Y | Y | Y | N | N | Y | Y | Y | 8/10 |
| Rucinski (2010) | Y | Y | Y | Y | Y | Y | Y | Y | Y | Y | 10/10 |
| Dinc & Sahin (2009) | Y | Y | Y | Y | Y | N | Y | Y | Y | Y | 9/10 |
| Fernandez et al (2003) | Y | Y | Y | Y | Y | Y | Y | Y | Y | Y | 10/10 |
| Sugarman et al (2002) | Y | Y | Y | Y | Y | N | Y | Y | Y | Y | 9/10 |
| Sugarman et al (1998) | Y | Y | Y | Y | Y | Y | Y | Y | Y | Y | 10/10 |

Y=Yes; N=No; U=Unclear

CASP. 2013. *Critical Appraisal Skills Programme (CASP): Making sense of evidence.* [Online]. <http://www.casp-uk.net/#!casp-tools-checklist/c18f8>. [Accessed April 30 2015].
